# A RESTful interface to pseudonymization services in modern web applications

**DOI:** 10.1186/s12911-014-0123-5

**Published:** 2015-02-07

**Authors:** Martin Lablans, Andreas Borg, Frank Ückert

**Affiliations:** Institute of Medical Biostatistics, Epidemiology and Informatics, University Medical Center of the Johannes Gutenberg University Mainz, Obere Zahlbacher Straße 69, Mainz, 55131 Germany

**Keywords:** Pseudonymization, Record linkage, Data protection, REST interface, Research network, Web application, Mainzelliste

## Abstract

**Background:**

Medical research networks rely on record linkage and pseudonymization to determine which records from different sources relate to the same patient. To establish informational separation of powers, the required identifying data are redirected to a trusted third party that has, in turn, no access to medical data. This pseudonymization service receives identifying data, compares them with a list of already reported patient records and replies with a (new or existing) pseudonym. We found existing solutions to be technically outdated, complex to implement or not suitable for internet-based research infrastructures. In this article, we propose a new RESTful pseudonymization interface tailored for use in web applications accessed by modern web browsers.

**Methods:**

The interface is modelled as a resource-oriented architecture, which is based on the representational state transfer (REST) architectural style. We translated typical use-cases into resources to be manipulated with well-known HTTP verbs. Patients can be re-identified in real-time by authorized users’ web browsers using temporary identifiers. We encourage the use of PID strings for pseudonyms and the EpiLink algorithm for record linkage. As a proof of concept, we developed a Java Servlet as reference implementation.

**Results:**

The following resources have been identified: Sessions allow data associated with a client to be stored beyond a single request while still maintaining statelessness. Tokens authorize for a specified action and thus allow the delegation of authentication. Patients are identified by one or more pseudonyms and carry identifying fields. Relying on HTTP calls alone, the interface is firewall-friendly. The reference implementation has proven to be production stable.

**Conclusion:**

The RESTful pseudonymization interface fits the requirements of web-based scenarios and allows building applications that make pseudonymization transparent to the user using ordinary web technology. The open-source reference implementation implements the web interface as well as a scientifically grounded algorithm to generate non-speaking pseudonyms.

## Background

Medical research networks collect data from separate, heterogeneous sources, such as disease registries, collections of biological material or hospital records. In order to conduct research on such data, one must be able to link records from different sources belonging to one patient. This poses two problems: First, unless all records carry a unique patient identifier, a record linkage algorithm is required to detect multiple occurrences of one patient based on their identifying information (name, date of birth etc.) while tolerating errors such as typos to a certain degree. Second, in order to protect the privacy of affected patients, the medical records must be pseudonymized before using them for research. This type of pseudonymization, which we call “first-level pseudonymization”, is to be distinguished from the case where an existing pseudonym is transformed into another, for example when patient identifiers in different domains need to be mapped to one another (see the discussion of PIX/PDQ in section ‘Related work’). In this paper, we are referring to first-level pseudonymization if not stated otherwise.

A common solution to the problem stated above is to delegate record linkage and pseudonymization to a trusted third party, called “first-level pseudonymization service”, which only stores the patients’ identifying data (“IDAT”) and creates a pseudonym, a random string by which medical data (“MDAT”) can be recognized as belonging to one patient without allowing re-identification. Nowadays, this transaction is frequently handled over a network connection on the internet (see the discussion of pseudonymization solutions in section ‘Related work’).

The typical data flow in this scenario is as follows: The data source, for example a hospital, transmits the identifying data of a patient to the pseudonymization service, which checks if the record already exists in its database. Depending on the outcome, either the existing pseudonym is returned or a new one is generated. After that, the data source exports the medical records of the patient together with the pseudonym to the researcher. This so-called informational separation of powers effectively protects privacy: To link medical information to an actual person, an attacker would have to gain access to both the IDAT and the MDAT database.

In clinical practice, however, it is impractical for physicians to retrieve patient records by a random string. Thus, if frequent re-identification is required, for example when data is also used for treatment of the affected patients, the technological infrastructure must provide a way to present IDAT along with MDAT to authorized users, rendering the informational separation of powers transparent.

### Aim

Our aim was to develop an interface for a first-level pseudonymization service that meets the following requirements often present in medical collaborative research:

- The interface is easily accessible by a wide range of source IT systems, especially web browsers.
- Support for non-speaking pseudonyms. Non-speaking means that a patient’s pseudonym is not functionally determined by her IDAT, as would be the case with hashing or encrypting it. This ensures that re-identification through the pseudonym alone is not possible. In addition, pseudonyms should be human-readable and representable in web application data and URLs.
- Support for transparent re-identification in the user’s web browser in scenarios where users need to see both IDAT and MDAT on the same webpage.
- Possibility to delegate user authentication to a different system in order to reduce administrative overhead. This is important because the pseudonymization service is usually run by a third party not necessarily involved in the research project or specialized in pseudonymization.
- Result is available to be used by third-party software implementations free of charge, and the possibility to enhance the interface definition and share such improvements with the research community and with software developers.

It must be clarified that our solution is limited to pseudonymization in the sense of replacing IDAT in the form of well-defined attributes. Related tasks like removing embedded IDAT from image data or free text forms, or preventing re-identification of patients through quasi-identifying attributes in the MDAT are research areas of their own and are not within the scope of this article. Also, our distinction between IDAT and MDAT does not preclude that the MDAT may contain some amount of demographic data (for example sex or age), provided that this does not significantly increase the risk of re-identification.

## Methods

### RESTful web services

REST (“Representational State Transfer”) was introduced as an architectural style for “distributed hypermedia systems” by Roy Fielding in his dissertation [1]. This style is the foundation of a class of web applications denoted as “RESTful”. However, as Fielding’s description is too abstract to translate directly into practical guidelines, developers of RESTful web services have relied on informal sources of information, leading to the situation of “REST’s best practices” being “a matter of folklore” [2] and to controversial discussions about what makes a web service “RESTful” or not (see, for example, the discussion on Fielding’s blog [3]).

A practical yet well-thought-out definition is given by Richardson and Ruby under the name “Resource-Oriented Architecture” [2]. Its core principles are:

- All interesting entities of the application (“resources”) are represented by descriptive URIs.
- Access to and manipulations of resources are handled by the standard HTTP methods (GET, POST, PUT, DELETE) and follow their semantics.
- The protocol is stateless in the sense that a request is independent from a previous request and includes all the information the server needs to understand it.

We base the interface of our pseudonymization service on these principles, as they fit its specific needs in several aspects.

First, because of its nature as a container, the patient list’s entities and operations translate naturally into resources and methods. For example, operations like “add a patient to the database” or “delete session 1234” are implemented by the HTTP requests POST/patients and DELETE/sessions/1234 (see section ‘Web interface’ for more examples).

Second, an interface based on pure HTTP makes the implementation of client applications easy and independent from the need for special libraries of frameworks: A patient list client can be programmed in any language or environment that is able to formulate HTTP requests, in particular web browsers.

Last but not least, being based on HTTP, this protocol is most firewall-friendly – a necessary requirement particularly in the medical domain, where strict network policies are common in order to protect sensitive data. Also, network connections can easily be protected using Transport Layer Security (SSL/TLS) to secure transmission of sensitive data via the proven HTTPs protocol.

Separating transport security from the interface allows administrators to freely choose reasonable security measures to fulfil the requirements of a specific application, ranging from encryption to more complex solutions like virtual private networks.

### Re-identification using temporary identifiers

In order to support re-identification of patients (i.e. display IDAT) in the user’s web browser or a similar user interface, we have implemented a data protection concept that was collaboratively created by members of the TMF, a German umbrella organization for medical research networks [4]. All sixteen data protection officers of the federal states in Germany have meanwhile agreed to this concept. A core requirement for use in applications related to patient treatment is the concept of temporary identifiers (“Temp IDs”). A Temp ID uniquely identifies a patient for the duration of a user session and is implemented as a hash value or a universally unique identifier (UUID) of sufficient length to prevent brute-force attacks. It is handed to the user’s web browser by the web server (hosted on the IDAT server in the original concept) and subsequently transmitted to the other server as an authorization ticket to obtain the missing data. This model obviates the need to authenticate the user on both the IDAT and the MDAT server and improves privacy as the permanent PID is not disclosed.

The first implementation of this concept in a web application was, to our knowledge, the teleradiological platform MDPE (*Medical Data and Picture Exchange*) [5], out of which the generic software library *DSLib* [6] evolved. DSLib facilitates the use of Temp IDs by supplying server-side functions for user sessions shared between two servers and JavaScript code for resolving Temp IDs (i.e. retrieving the corresponding data) via asynchronous requests (AJAX). It also offers solutions to problems arising from the same origin policy [7], which poses problems in the event that MDAT and IDAT servers are run under different hostnames. We used the model of the DSLib, especially the concept of Temp IDs, as building blocks for supporting re-identification in our pseudonymization interface. However, in contrast to the TMF concept, we assume that user authentication and the web site are hosted together with MDAT and that IDAT is loaded via Temp IDs. This has proven more suitable in practice as IDAT are most often handed by a trusted third party not directly involved in the research project (as already noted in the list of aims).

### Optimal patient identifiers

For use as a pseudonym, Faldum and Pommerening have proposed an “optimal code for patient identifiers” [8]. A “PID” is a string of eight characters from an alphabet of numbers and uppercase letters, excluding easily confusable entries such as “BIOS”/”8105”. Generation happens deterministically by encrypting a counter that is incremented for every new entry. The encryption algorithm uses three secret keys stored in the PID generator’s configuration. This procedure ensures that a PID contains neither information on the original IDAT nor on the order of generation, i.e. two instances with different values for the keys produce PIDs in a different order even if the same data is entered. Faldum and Pommerening have further shown that in a given PID string, up to two errors can be detected and one erroneous character as well as the transposition of two adjacent characters can be corrected.

Although the presented interface does not enforce the use of PID strings as pseudonyms, we encourage its use due to the stated reasons. Regarding the use in web applications, the brevity of the PID string and its limitation to ASCII letters and numbers support its handling in URLs and text-encoded data.

### Record linkage

In general, record linkage is the process that determines which entries in a set of personal records belong to one person. In the case of a pseudonymization framework, record linkage ensures that multiple requests for the same personal record return the same pseudonym, ideally even when slight discrepancies such as spelling errors occur (disregarding use cases where multiple pseudonyms for one person exist within different domains). See Table 1 for an example.

**Table 1:** **Exemplary record linkage dataset**

**First name**	**Last name**	**Date of birth**
Ivan Peter	Fellegi	1935-06-22
Ivan	Felligi	1935-06-22

There is considerable research in designing and improving record linkage algorithms, for an overview, see [9,10]. The record linkage process in itself is not part of the pseudonymization interface and thus out the of scope of this article. However, the ability to identify patients across multiple data sources is an essential motivation to delegate pseudonymization to a centralized service instead of generating pseudonyms locally. We therefore recommend that every implementation of the interface provide a reasonable record linkage algorithm.

### Development process

Having chosen the resource-oriented architecture pattern, we translated the use-cases (outlined in the introduction) into resources and methods. As a proof of concept, we developed a Java implementation of the resulting interface: Starting with a top-down approach, we implemented resources and methods using the Jersey framework for RESTful web services [11]. In a following bottom-up phase, we derived the required backend elements such as persistency methods and data classes from the resources and methods and implemented them. Lastly, we added the code for serving requests and record linkage.

## Results

### Web interface

In the most common use case of a web-based medical registry, three parties are involved in a request to the patient list. Apart from the patient list itself, this includes the user who wants to retrieve a PID for a patient, and the MDAT server, which also hosts the HTML interface. As shown in the following, the MDAT server serves as a kind of intermediary between the human user and the patient list.

#### Resources

In the following, we first introduce the most important resources of the patient list’s interface. Then we demonstrate its usage by an example.

##### Sessions (/sessions)

Similar to a browser’s session with a web server, a session in the patient list allows data associated with a client to be stored beyond a single request. In order to maintain compatibility with the REST paradigm’s property of statelessness (compare section ‘The RESTfulness of the patient list interface’), a session is modeled as a resource /sessions/{sessionid}, where {sessionid} is a unique identifier. Data items belonging to a session are modeled as subordinate resources, tokens (as described hereinafter) being the most important ones.

Sessions are created and managed by the MDAT server. It is advisable that the MDAT server mirror every browser session with a session on the patient list server, although this cannot be enforced due to the fact that authentication of individual users is a duty of the MDAT server (see bullet point “Possibility to delegate user authentication” in section ‘Aim’). Mapping sessions to users can easily be implemented by creating a session once a user logs in and storing the session id in the user account data.. Once the user logs out, the MDAT server only has to delete the corresponding session resource in order to invalidate all tokens associated with that session (see the next section on tokens).

##### Tokens (/sessions/{sessionid}/tokens)

A token represents the authorization to perform a specified action in the patient list, for example to request a PID for a new patient. As expressed by the resource path, every token belongs to one session. Tokens are usually handed to the user’s web browser by the MDAT server. No other authentication is required other than knowing the token identifier, which is a random UUID as proposed in RFC 4122 [12].

##### Patients (/patients)

Obviously, patients are the most important entities in a patient list, identified by a PID. A PID request is modeled as a POST request to this resource. The request bears a set of identifying attributes.

#### Example communication

**Figure 1:**
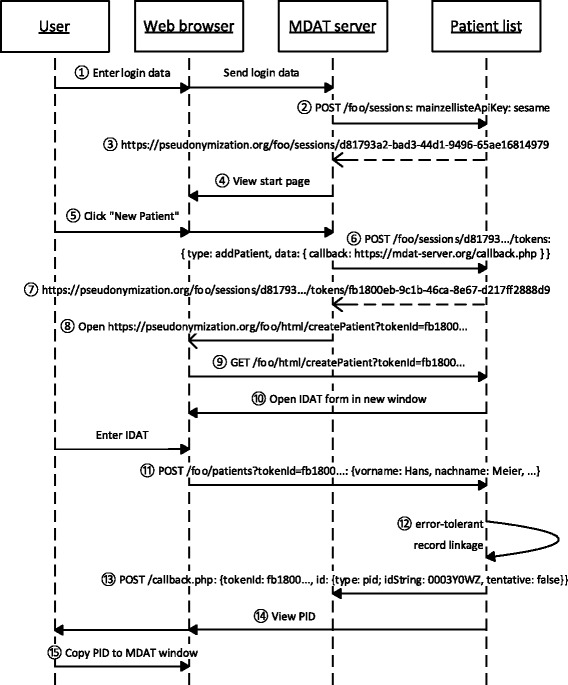
**Example communication between user, web browser, MDAT server and pseudonymization service (IDAT server).**

Figure 1 shows an example communication in which a pseudonym for a patient is retrieved in order to enter medical data into a pseudonymized registry. We assume that the pseudonymization service is available at https://pseudonymization.org/foo and is accessed by the MDAT server with the fully qualified domain name mdat-server.org. The pseudonymization process is initiated by a user through her web browser. Both the web browser and the MDAT server act as clients of the patient list.

First, the user logs into the registry system with her credentials ①. After successful authentication, the MDAT server registers a session on the IDAT server through a POST request to the /sessions resource ②. The MDAT server authenticates itself against the patient list by supplying a secret key, “sesame” in this example, in the HTTP header mainzellisteApiKey^a^. A new session is created and its URL is returned ③. This session is associated with the browser session between the user’s web browser and the MDAT server, although the web browser never accesses it directly. The MDAT server now completes its own initialization of the user session and returns the start page of the registry application ④.

Next, we assume that the user wishes to enter medical data of a new patient. She clicks on a button or link that the MDAT application provides for requesting a new PID ⑤. The MDAT server now creates a token by a POST request to the tokens resource within the active session (/sessions/{session-id}/tokens, ⑥). Supplied with the request is a JSON object with the components type and data. The token type is a string that defines for what action the token is valid. In this case, the token type is addPatient, i.e. the token allows a patient to be added to the list. The data component of the token is another JSON object where, depending on the token type, specific key-value pairs can be stored. For an addPatient token, a callback address can be supplied, to which the patient list posts a request after a successful PID request has been made with the token. The purpose of the callback is that the MDAT server is notified of the result of a PID request in advance of the user’s web browser. It can then perform initializations such as creating a medical record for a new patient or redirecting the user to the requested patient.

Having received the new token ⑦, the MDAT server redirects the user to an HTML page on the IDAT server, which provides a form to enter IDAT for a PID request (⑧, ⑨, ⑩). The token identifier is added as a URL parameter for authorization. In this example, it is assumed that the form is opened in a new browser window, but principally it could be embedded in an IFrame. The form is submitted by a POST request to the /patients resource, again with the token identifier added for authorization . The IDAT server now utilizes its record linkage component to decide whether to return an existing or a new PID . The MDAT server is notified of the result through the callback address stored in the token , and the PID is presented to the user in the IDAT window . She can now copy it to the MDAT form  and proceed with entering the patient’s MDAT. As a convenient alternative, the user’s web browser can be referred to an arbitrary page on the MDAT server by supplying a redirect URL upon creating the token.

The Temp IDs used for re-identification in the user’s web browser are realized by another token type, readPatients, thereby omitting the need of an additional data structure. Like the addPatient token, it is created by a request from the MDAT server to the tokens resource. The token identifier can then be embedded in the HTML output of the MDAT server and resolved in the web browser by JavaScript code. Another use case that can be implemented by this token type is the transformation of one pseudonym into another (“second-level pseudonymization”). For this purpose, the token can be configured to allow reading pseudonyms of a specified type (i.e. domain).

### Reference implementation

**Figure 2:**
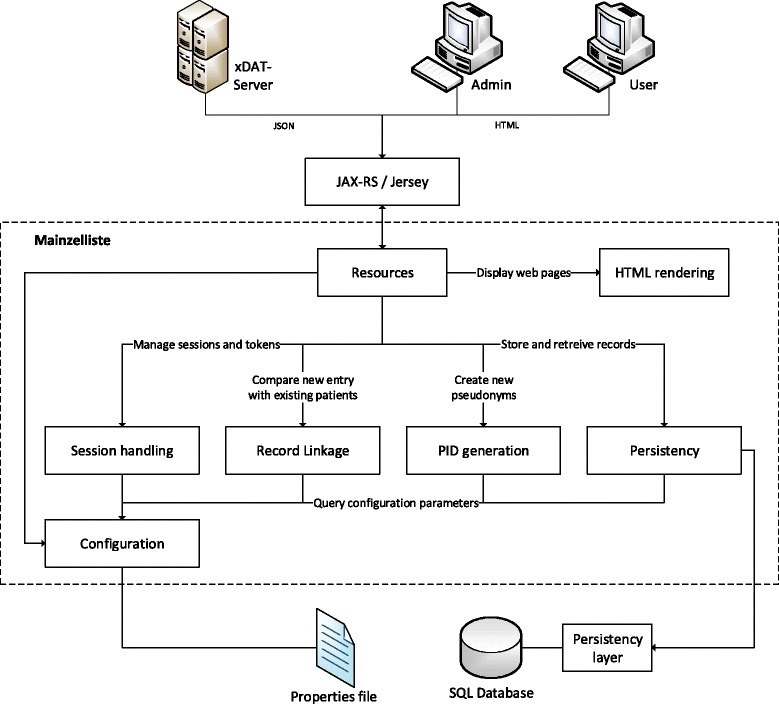
**Components of the Mainzelliste reference implementation.** The arrows indicate in which direction components of the application access each other. See section ‘Reference implementation’ for a description of the components and their relations.

As proof of concept and reference implementation, we have developed “Mainzelliste”. Figure 2 illustrates its general components and its interfaces to the outside world: Clients (i.e. other servers, the administrator and users) connect to the application via the described REST interface. A “Resources” component provides methods to which the incoming requests are mapped by the use of JAX-RS annotations. It then uses the following backend components to fulfill the request:

- Session handling: Provides a (non-persistent) container to store sessions and tokens and provides methods to access them.
- HTML rendering: Utilizes JavaServer Pages to render the HTML interface for admin and user access.
- Matching: Contains the classes that are involved in the record linkage, including preprocessing of incoming fields.
- PID generation: Provides generation and verification functions for ID strings, such as the algorithm by Faldum and Pommerening [8].
- Persistency: Provides high-level methods to save and retrieve data to and from the database, such as “get a list of all patients” or “add patient (…) to the list”. The persistency component accesses a relational database through a persistency layer, which handles the necessary object-relational mapping.
- Configuration: This singleton object provides access to the application’s configuration read from a properties file at startup. All other components retrieve their specific configuration properties from this component.

Mainzelliste satisfies the functional requirements stated in the introduction: It generates non-speaking pseudonyms, supports re-identification in a web browser and offers all functions through the described RESTful web service. Its record linkage implementation is based on the “Epilink” algorithm proposed by Contiero et al. [13].

Mainzelliste has proven to be production stable and has been released as open-source software to be freely used, reverse-engineered and modified by any interested party.

## Discussion

In order to discuss the advantages and shortcomings of our interface, we compare the reference implementation described in the preceding section to similar software applications.

### Related work

#### PID generator: interface and compatibility

In order to accommodate the needs of research communities for a first-level pseudonymization tool, Pommerening et al. designed the PID generator [14]. Its main features are a rule-based record linkage algorithm and the creation of non-speaking pseudonyms via the algorithm by Faldum and Pommerening (see section ‘Optimal patient identifiers’). The PID generator and its drawbacks were a major motivation for developing Mainzelliste as a possible replacement.

Since its initial release in 2003, the PID generator has been in use by 10-20 networks (a figure estimated in 2012 by the TMF office, its main distributor), rendering it a de-facto standard for German networks. However, having aged with little improvements, we do not deem the PID generator suitable for a state-of-the-art implementation of research infrastructure anymore. This verdict is for two reasons: First, the original PID generator offers only a command-line interface, meaning that network access has to be provided by an additional layer [15] using software components that have meanwhile become outdated. Second, its inability to handle Unicode characters and its incompatibility with 64-bit systems pose difficulties for the application in an international context and in combination with current computing infrastructure. More recent implementations such as those presented here overcome these restrictions through their web interface and their use of Java, thus providing compatibility with a variety of platforms.

#### IHE PIX/PDQ

While a patient list as outlined in this paper is to some extent comparable to a PIX/PDQ provider, there are subtle but important differences. Concerning the overall structure, the PIX profile is based on connecting several domains (like departments or different software systems of a hospital) in which patients are already uniquely identified. The central component that links the domain-specific identifiers of a patient (“Patient Identifier Cross-reference Manager”) receives patient data not from arbitrary sources, but from only one specific data provider (“Patient Identity Source Actor”) for each domain [16]. In the case of a research network, however, there is often only one domain (i.e. each patient is assigned a single, network-wide patient identifier), but requests to enter patient data come from a large number of users.

In addition to these structural differences, typical data flows of a research network do not translate easily into PIX/PDQ transactions. Consider, for example, the case of supplying identifying data and retrieving a pseudonym. The transaction “Patient Identity Feed”, by which a patient is registered at the PIX provider, does not return a result and requires an existing patient identifier to be transmitted in the request. To retrieve patient identifiers (i.e. pseudonyms), PIX/PDQ provides “PIX query”, but this transaction does not accept IDAT; rather, it requires the sender to provide an existing patient identifier. Relatively speaking, the best suitable option for the mentioned use case (retrieving a pseudonym for a patient) is the “Patient Demographics Query” transaction, which accepts identifying data as search criteria. Utilizing PDQ as a pseudonymization function, however, would require behavior not expected from its specification and normal use: First, PDQ is meant as a read-only query, but pseudonymizing a new patient would require creating a new patient record and identifier. Second, PDQ allows a list of patients to be retreived when providing incomplete data (e.g. last name only) – the use for pseudonymization would require deactivating this functionality, thereby violating the specification.

#### Open EMPI: REST-RPC Hybrid vs. “true” REST

OpenEMPI (Enterprise Master Patient Index) is an open-source application that provides a central patient registry [17]. It implements the IHE profiles “Patient Identity Cross-referencing” (PIX) and “Patient Demographics Query” (PDQ) and shares their drawbacks mentioned in the previous paragraph, making it more a candidate for integration in a clinical context (e.g. a clinical data warehouse) than for a distributed research project. The interface of OpenEMPI, although described as being RESTful, is in fact of the style denoted as “REST-RPC-Hybrid” by Richardson and Ruby [2] – remote procedure calls embedded in a resource-based interface. Specifically, OpenEMPI utilizes resources to represent functions that could simply be modeled by standard HTTP methods. For example, removing a record is not modeled as a DELETE on a resource that represents the patient, but as a PUT on a resource that represents the function (/person-manager-resource/deletePerson), with the data necessary to identify the patient attached in the request body [17]. This is a reasonable approach to building a web interface, but the result is considerably less intuitive and self-explanatory than a true RESTful interface. In practice, developers who program client applications will have to consult the interface documentation more often if every function is modeled by a resource than if well-known HTTP methods are utilized.

#### E-PIX: “Big Web Service” vs. REST

At the university of Greifswald, Schack et al. have developed the pseudonymization tool E-PIX, implemented as a Java EE compliant application, for use within their research project GANI_MED (Greifswald Approach to Individualized Medicine) [18]. In contrast to Mainzelliste, whose REST interface sticks with the capabilities provided by HTTP, E-PIX utilizes additional layers, namely SOAP and WS-Security, to understand and process requests. We agree with Richardson and Ruby that the complexity of such “Big Web Services” ties up resources better put into features and performance – concerning the development of the software itself as well as of clients connecting to it [2]. However, one must keep in mind that E-PIX was initially developed for use in an application where integration into the existing clinical IT infrastructure was important and no significant network limitations (bandwidth, latency) had to be considered.

In contrast to our design, E-PIX handles user roles and authorization as part of the IDAT application. As stated before, we deem that impractical for the frequent case that a pseudonymization service is run by external providers. Once again, the different approach of E-PIX is motivated by its use in a clinical environment and seems reasonable for the originally intended clinical use case.

The developers of E-PIX have integrated the Mainzelliste interface into E-PIX. As a result, E-PIX can be integrated in a clinical context (due to its conformance to relevant IHE profiles) as well as in web-based research network applications.

### The RESTfulness of the patient list interface

Having criticized the hybrid REST interface style of OpenEMPI, it is worth questioning whether our pseudonymization interface is truly “RESTful”. In fact, there is one arguable point: The statelessness of the protocol. Fielding describes statelessness as the fact that “each request from client to server must contain all of the information necessary to understand the request, and cannot take advantage of any stored context on the server” [1]. In Richardson’s and Ruby’s terms, statelessness means that “[t]he server never relies on information from previous requests” [2]. In case of the patient list, the fact that access to a session relies on that session being created beforehand looks like a violation of this requirement: A POST to /sessions/abc/tokens can only be successful if a session named “abc” has been created by a previous POST /sessions, and the server has to memorize the result of the first request to serve the second. However, this argument can be refuted by an alternative view: Creating a token does not rely on the specific request by precisely this client, but on the sole existence of the session. After all, the session is not private between one client and the server (similar to the working directory in an FTP session), but disclosed as a resource with its own URI. In fact, any other client could access the session, provided that he knows its URI and has a valid access key. Therefore, the existence of the session falls into the category that Richardson and Ruby denote as “resource state”, meaning state information that “is the same for each client”; they argue that this kind of state information is exempted from the postulation of statelessness.

The question as to what extend the patient list operates statelessly is not purely academic, but a question of flexibility: Thanks to the statelessness of the protocol, there are few restrictions on the order in which requests can be made. For example, a client can choose based on the given application’s requirements to either first create ten sessions, and thereafter one token in each of them, or to instantiate sessions and their associated tokens alternately – in terms of Richardson and Ruby, the client itself is “in charge of managing its own path through the application” [2].

### Distribution and dissemination

The described RESTful pseudonymization interface has, in addition to the reference implementation, already been implemented by another pseudonymization service (E-PIX, see section ‘E-PIX: “Big Web Service” vs. REST’), a widely used commercial electronic data capture system [19], and, to a large extent, by the German National Cohort [20,21]. Also, Skripcak et al. have used the interface with OpenClinica as part of a platform for radiotherapy [22].

The Mainzelliste reference implementation is, in addition to several of our own projects [23-25], already in production use for a European-wide web-based registry for childhood interstitial lung diseases [26]. Further usage metrics are hard to obtain due to its release as AGPL-licensed software that is free to download and redistribute. The BitBucket repository has been forked six times and we receive enhancement requests and various feedback on a mailing list consisting of thirteen sites located in Switzerland and Germany.

However, dissemination currently seems to stop at the German linguistic border. As a consequential next step, we aim to extend further development of the interface as well as its reference implementation to the international scientific community and its software developers. To do so, we are currently establishing thorough English technical documentation, a bug tracker to process change requests, and a system for backward compatibility.

## Conclusion

We have presented a RESTful pseudonymization interface that fits the requirements of web-based applications in medical research networks. Apart from first-level pseudonymization and record linkage, it supports transparent re-identification and delegation of authentication. Thanks to being RESTful, it is firewall-friendly, flexible and easily implemented. Integrated into two patient list applications and a widely used commercial EDC system, the interface has gained somewhat wide distribution.

We have also provided a reference implementation comprising a flexible record linkage framework and a scientifically grounded algorithm to generate non-speaking pseudonyms. Mainzelliste is suited for production environments and available as free software under the GNU Affero General Public License (AGPL version 3 or later) at http://www.mainzelliste.de, with its source code available in a public repository at https://bitbucket.org/medinfo_mainz/mainzelliste.

## Endnote

^a^ The naming of the parameter relates to the reference implementation “Mainzelliste”, see section ‘Reference implementation’.
